# Supplementary figures and images for: Neuroprotective effects of TRPA1 channels in the cerebral endothelium following ischemic stroke
Source: eLife. 2018 Sep 21;7:e35316. doi: 10.7554/eLife.35316 (PMC6177258; doi:10.7554/eLife.35316)

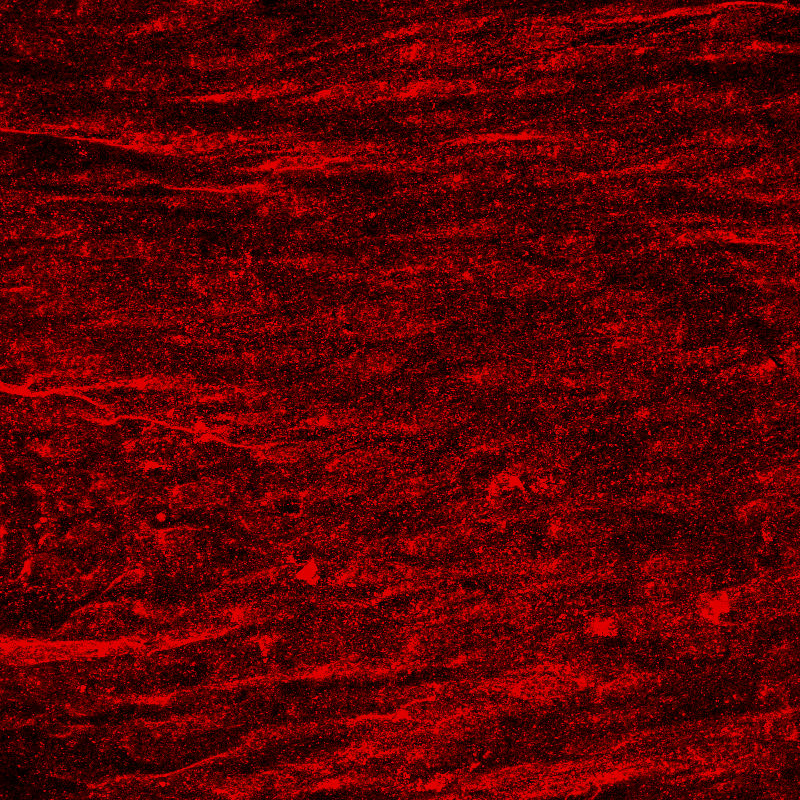

Supplement: Figure 3—figure supplement 1—source data 1. [file elife-35316-fig3-figsupp1-data1.zip › Figure 3 Figure supplement 1 Max Intensity RGB Images/Hypoxia ROI_01_4HNE channel_RGB.tif]

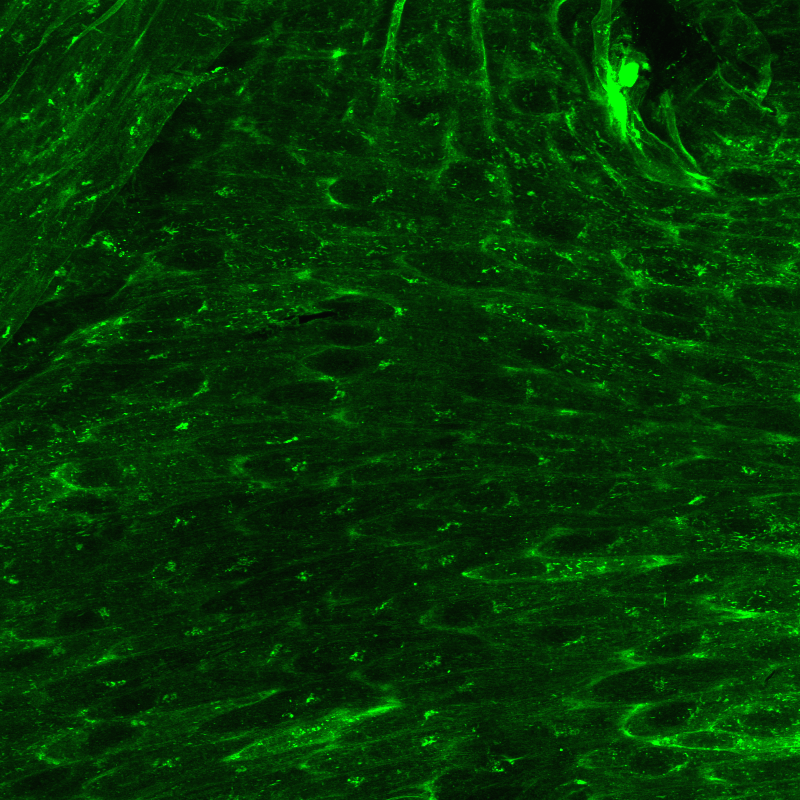

Supplement: Figure 3—figure supplement 1—source data 1. [file elife-35316-fig3-figsupp1-data1.zip › Figure 3 Figure supplement 1 Max Intensity RGB Images/Normoxia ROI_02_Isolectin channel.tif]

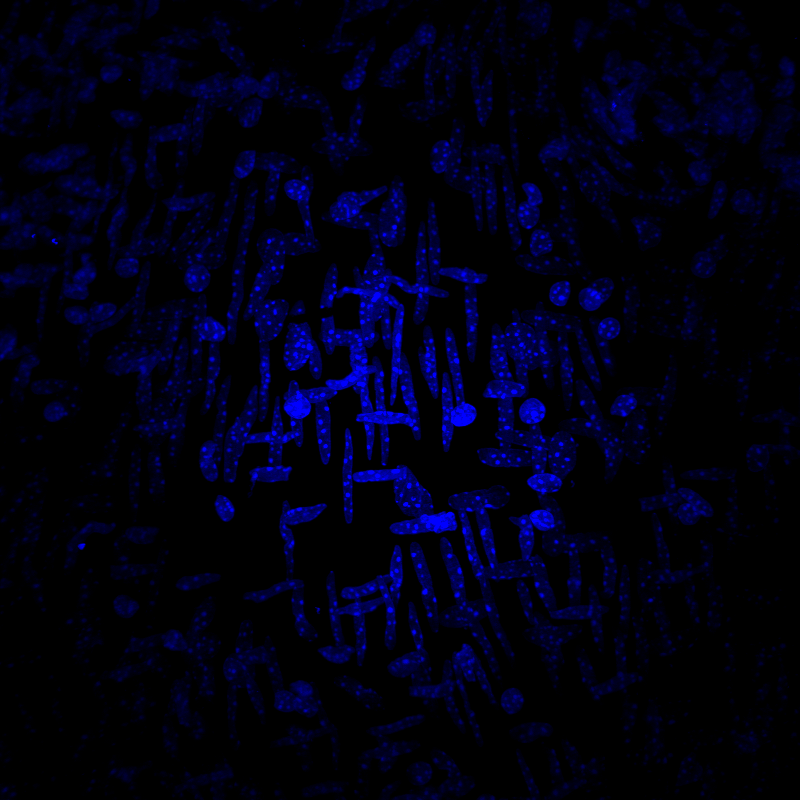

Supplement: Figure 3—figure supplement 1—source data 1. [file elife-35316-fig3-figsupp1-data1.zip › Figure 3 Figure supplement 1 Max Intensity RGB Images/Normoxia ROI_02_DAPI channel.tif]

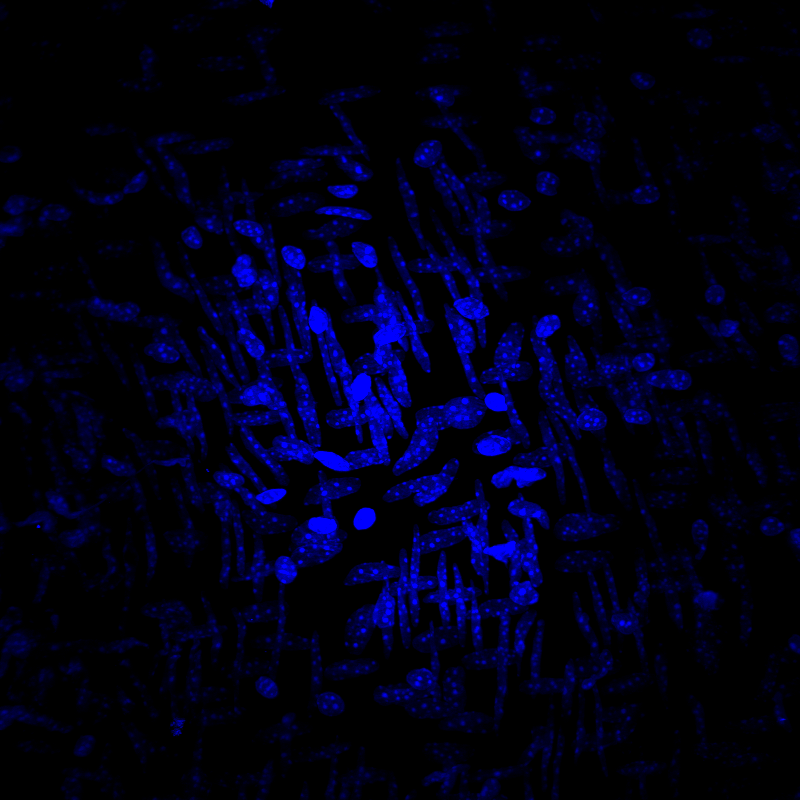

Supplement: Figure 3—figure supplement 1—source data 1. [file elife-35316-fig3-figsupp1-data1.zip › Figure 3 Figure supplement 1 Max Intensity RGB Images/Hypoxia ROI_01_DAPI channel_RGB.tif]

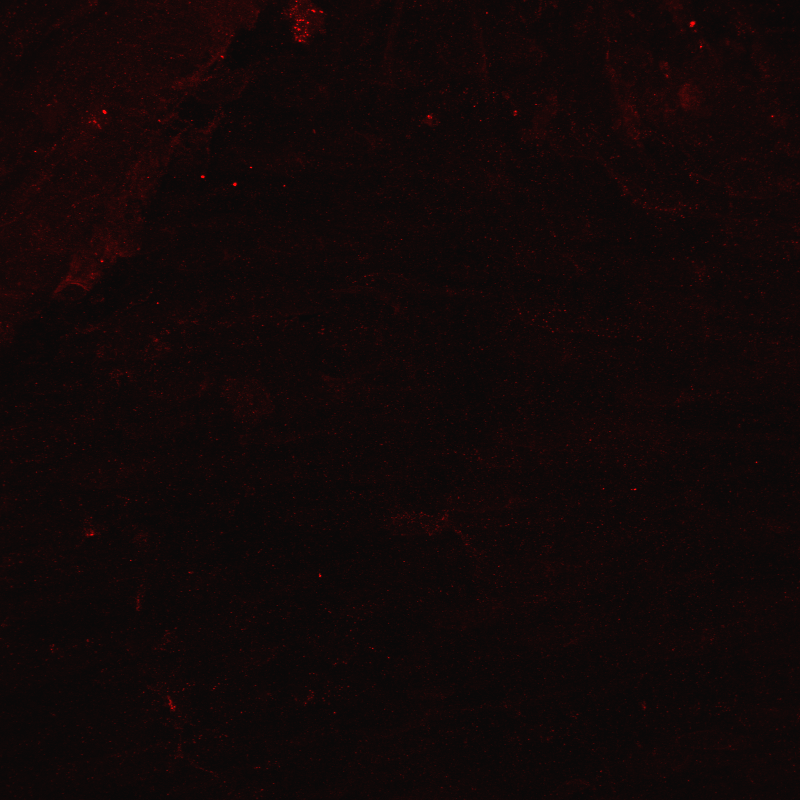

Supplement: Figure 3—figure supplement 1—source data 1. [file elife-35316-fig3-figsupp1-data1.zip › Figure 3 Figure supplement 1 Max Intensity RGB Images/Normoxia ROI_02_4HNE channel.tif]

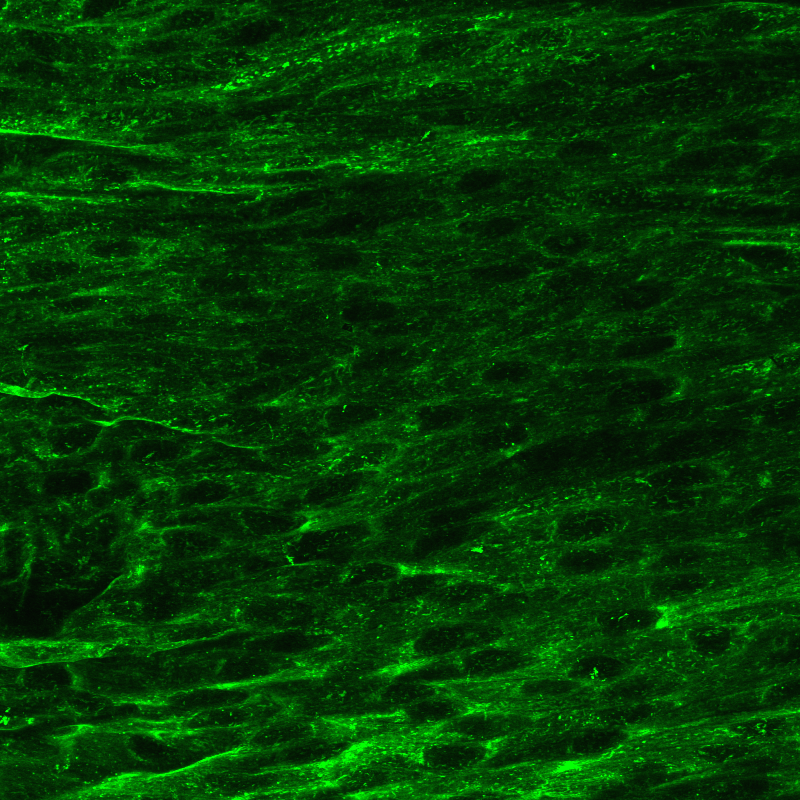

Supplement: Figure 3—figure supplement 1—source data 1. [file elife-35316-fig3-figsupp1-data1.zip › Figure 3 Figure supplement 1 Max Intensity RGB Images/Hypoxia ROI_01_Isolectin channel_RGB.tif]
