# Supplementary material for: Neuroprotective effects of TRPA1 channels in the cerebral endothelium following ischemic stroke
Source: eLife. 2018 Sep 21;7:e35316. doi: 10.7554/eLife.35316 (PMC6177258; doi:10.7554/eLife.35316)
Supplement: Supplementary file 1. [file elife-35316-supp1.docx]

**Supplemental File 1.** Properties of TRPA1 sparklets induced by 4-hydroxynonenal (4-HNE) and hypoxia.

|  | **4-HNE** | **Hypoxia** |
| --- | --- | --- |
| *Mode Amplitude (ΔF/F_0_)* | 1.10 | 1.10 |
| *Mode Duration (ms)* | 240 | 240 |
| *Mean Duration (ms)* | 450 ± 0.017 | 397 ± 0.001 |
| *Mode Spatial Spread (μm^2^)* | 10 | 12 |
| *Mean Spatial Spread (μm^2^)* | 17.2 ± 0.4 | 19.8 ± 0.5 |
