# Supplementary material for: Neuroprotective effects of TRPA1 channels in the cerebral endothelium following ischemic stroke
Source: eLife. 2018 Sep 21;7:e35316. doi: 10.7554/eLife.35316 (PMC6177258; doi:10.7554/eLife.35316)
Supplement: Supplementary file 2. [file elife-35316-supp2.docx]

**Supplemental File 2.** Properties of large Ca^2+^ transients in the cerebral endothelium.

|  | **Vehicle** | **4-HNE** |
| --- | --- | --- |
| *Mode Amplitude (ΔF/F_0_)* | 1.10 | 1.10 |
| *Mode Duration (ms)* | 500 | 250 |
| *Mean Duration (s)* | 3.01 ± 0.12 | 1.62 ± 0.05 |
| *Mode Spatial Spread (μm^2^)* | 20 | 20 |
| *Mean Spatial Spread (μm^2^)* | 46.9 ± 2.5 | 39.9 ± 1.3 |
